# Supplementary material for: A user preference analysis of commercial breath ketone sensors to inform the development of portable breath ketone sensors for diabetes management in young people
Source: PLoS One. 2022 Jul 25;17(7):e0269925. doi: 10.1371/journal.pone.0269925 (PMC9312428; doi:10.1371/journal.pone.0269925)
Supplement: S5 Appendix — (DOCX) [file pone.0269925.s005.docx]

**Supplementary material**

Breath and Blood Measurements in the Sample

| IP | Device 1 | Day | Time | Breath ketone (mg/l) | Blood ketone (mmol/l) | Blood glucose (mmol/l) | Unwell | Comments |
| --- | --- | --- | --- | --- | --- | --- | --- | --- |
| 1 | KetoPro KHC M3 | Day 1 | before breakfast | 0.08 | 0.10 | 6.90 | No | Slight sore throat |
|  |  |  | after school (before eating) | 0.00 | 0.10 | 14.80 | No |  |
|  |  | Day 2 | before breakfast | 0.11 | 0.10 | 10.80 | No | Slight sore throat |
|  |  |  | after school (before eating) | 0.00 | 0.20 | 12.00 | No |  |
|  |  | Day 3 | before breakfast | 0.08 | 0.40 | 9.50 | x | Wondering if the toothpaste used will affect the reading? |
|  |  |  | after school (before eating) | 0.00 | 0.01 | 11.80 | x |  |
|  |  | Day 4 | before breakfast | 0.06 | 0.00 | 9.40 | x |  |
|  |  |  | after school (before eating) | 0.00 | 0.01 | 6.20 | x |  |
|  |  | Day 5 | before breakfast | 0.00 | 0.01 | 9.70 | x | Later times today as it’s a Saturday |
|  |  |  | after school (before eating) | 0.00 | 0.10 | 8.80 | x |  |
| 2 | KetoPro KHC M3 | Day 1 | before breakfast | 0.66 | 0.20 | x | No |  |
|  |  |  | after school (before eating) | 0.66 | 0.10 | x | No |  |
|  |  | Day 2 | before breakfast | 0.00 | 0.60 | x | Yes | Cold/flu |
|  |  |  | after school (before eating) | 0.00 | 0.20 | x | Yes | Cold/flu |
|  |  | Day 3 | before breakfast | 0.00 | 0.00 | x | No |  |
|  |  |  | after school (before eating) | 0.00 | 0.00 | x | No |  |
|  |  | Day 4 | before breakfast | 0.00 | 0.10 | x | No |  |
|  |  |  | after school (before eating) | 0.00 | 0.00 | x | No |  |
|  |  | Day 5 | before breakfast | 0.00 | 0.10 | x | No |  |
|  |  |  | after school (before eating) | 0.00 | 0.00 | x | No |  |
| 3 | KetoPro KHC M3 | Day 1 | before breakfast | 0.00 | 0.20 | 5.90 | No |  |
|  |  |  | after school (before eating) | 0.00 | 0.00 | 4.80 | No |  |
|  |  | Day 2 | before breakfast | 0.00 | 0.10 | 9.30 | No |  |
|  |  |  | after school (before eating) | 0.00 | 0.10 | 3.60 | No |  |
|  |  | Day 3 | before breakfast | 0.00 | 0.10 | 7.20 | No |  |
|  |  |  | after school (before eating) | 0.00 | 0.10 | 5.90 | No |  |
|  |  | Day 4 | before breakfast | 0.00 | 0.10 | 5.10 | No |  |
|  |  |  | after school (before eating) | 0.00 | 0.20 | 12.10 | No |  |
|  |  | Day 5 | before breakfast | 0.00 | 0.10 | 6.40 | No |  |
|  |  |  | after school (before eating) | 0.00 | 0.00 | 4.90 | No |  |
|  |  | Day 6 | before breakfast | 0.00 | 0.10 | 4.90 | No |  |
|  |  |  | after school (before eating) | 0.00 | 0.20 | 7.80 | No |  |
|  |  | Day 7 | before breakfast | 0.00 | 0.00 | 6.60 | No |  |
|  |  |  | after school (before eating) | 0.00 | 0.20 | 10.20 | No |  |
|  |  | Day 8 | before breakfast | 0.00 | 0.10 | 5.50 | No |  |
|  |  |  | after school (before eating) | 0.00 | 0.00 | 4.20 | No |  |
|  |  | Day 9 | before breakfast | 0.00 | 0.00 | 6.40 | No |  |
|  |  |  | after school (before eating) | 0.00 | 0.10 | 7.90 | No |  |
| 4 | KetoPro KHC M3 | Day 1 | before breakfast | 0.00 | 0.00 | 9.60 | No |  |
|  |  |  | after school (before eating) | 0.01 | 0.10 | 17.40 | No |  |
|  |  | Day 2 | before breakfast | 0.01 | 0.00 | 8.20 | No |  |
|  |  |  | after school (before eating) | 0.00 | 0.00 | 12.70 | No |  |
|  |  | Day 3 | before breakfast | 0.02 | 0.10 | 5.30 | No |  |
|  |  |  | after school (before eating) | 0.01 | 0.10 | 8.30 | No |  |
|  |  | Day 4 | before breakfast | 0.00 | 0.10 | 15.10 | No |  |
|  |  |  | after school (before eating) | 0.00 | 0.00 | 10.40 | No |  |
|  |  | Day 5 | before breakfast | 0.01 | 0.00 | 16.50 | No |  |
|  |  |  | after school (before eating) | 0.00 | 0.00 | 13.60 | No |  |

| IP | Device 2 | Day | Time | Breath ketone (g/l) | Blood ketone (mmol/l) | Blood glucose (mmol/l) | Unwell | Comments |
| --- | --- | --- | --- | --- | --- | --- | --- | --- |
| 5 | House of Keto | Day 1 | before breakfast | 0.00 | 0.00 | 8.40 | No |  |
|  |  |  | after school (before eating) | 0.00 | 0.00 | 7.60 | No |  |
|  |  | Day 2 | before breakfast | 0.00 | 0.10 | 5.10 | No |  |
|  |  |  | after school (before eating) | 0.00 | 0.10 | 3.90 | No |  |
|  |  | Day 3 | before breakfast | 0.00 | 0.10 | 7.60 | No |  |
|  |  |  | after school (before eating) | 0.00 | 0.10 | 4.10 | No |  |
|  |  | Day 4 | before breakfast | 0.00 | 0.10 | 8.30 | No |  |
|  |  |  | after school (before eating) | 0.00 | 0.10 | 7.20 | No |  |
|  |  | Day 5 | before breakfast | 0.00 | 0.10 | 6.60 | No |  |
|  |  |  | after school (before eating) | 0.00 | 0.10 | 9.10 | No |  |
| 6 | House of Keto | Day 1 | before breakfast | 0.00 | 0.00 | 12.30 | No |  |
|  |  |  | after school (before eating) | 0.00 | 0.10 | 10.50 | No |  |
|  |  | Day 2 | before breakfast | 0.00 | 0.10 | 12.80 | No |  |
|  |  |  | after school (before eating) | 0.00 | 0.10 | 14.10 | No |  |
|  |  | Day 3 | before breakfast | 0.00 | 0.10 | 6.70 | No |  |
|  |  |  | after school (before eating) | x | x | x | x |  |
|  |  | Day 4 | before breakfast | 0.00 | 0.00 | 12.20 | No |  |
|  |  |  | after school (before eating) | 0.00 | 0.10 | 16.40 |  |  |
|  |  | Day 5 | before breakfast | 0.00 | 0.00 | 8.60 | No |  |
|  |  |  | after school (before eating) | 0.00 | 0.10 | 13.30 | No |  |
|  |  | Day 6 | before breakfast | 0.00 | 0.10 | 11.30 | No |  |
|  |  |  | before eating | 0.00 | 0.30 | 7.30 | No | I had something to eat and tested 20 minutes after I had forgotten to test first |
|  |  | Day 7 | before breakfast | 0.00 | 0.10 | 11.20 | No |  |
|  |  |  | before eating | 0.00 | 0.10 | 20.00 | No | High when testing so performed a ketone reading then |
|  |  | Day 8 | before breakfast | No reading | No reading | 7.30 | No |  |
|  |  |  | after school (before eating) | 0.00 | 0.10 | 13.50 | No |  |
|  |  | Day 9 | before breakfast | 0.00 | 0.00 | 5.10 | No |  |
|  |  |  | after school (before eating) | 0.00 | 0.10 | 12.70 | No |  |
| 7 | House of Keto | Day 1 | before breakfast | 0.00 | 0.10 | 11.00 | No | Generally well |
|  |  |  | after school (before eating) | 0.00 | 0.10 | 7.10 | No | Generally well |
|  |  | Day 2 | before breakfast | 0.00 | 0.10 | 6.90 | No | Generally well |
|  |  |  | after school (before eating) | 0.00 | 0.10 | 15.00 | No | Generally well |
|  |  | Day 3 | before breakfast | 0.00 | 0.10 | 5.70 | No | Generally well |
|  |  |  | after school (before eating) | 0.00 | 0.10 | 14.10 | No | Generally well |
|  |  | Day 4 | before breakfast | 0.00 | 0.10 | 13.00 | No | Generally well |
|  |  |  | after school (before eating) | 0.00 | 0.10 | 2.80 | No | Generally well |
|  |  | Day 5 | before breakfast | 0.20 | 0.10 | 11.60 | No | Well |
|  |  |  | after school (before eating) | 0.00 | 0.10 | 5.20 | No | Well |
| 8 | House of Keto | Day 1 | before breakfast | 0.00 | 0.00 | 4.00 | No |  |
|  |  |  | after school (before eating) | 0.01 | 0.10 | 8.60 | No |  |
|  |  | Day 2 | before breakfast | 0.00 | 0.30 | 7.70 | No |  |
|  |  |  | after school (before eating) | 0.00 | 0.10 | 7.80 | No |  |
|  |  | Day 3 | before breakfast | 0.00 | 0.10 | 7.10 | No |  |
|  |  |  | after school (before eating) | 0.00 | 0.10 | 5.70 | No |  |
|  |  | Day 4 | before breakfast | 0.00 | 0.10 | 8.80 | No |  |
|  |  |  | after school (before eating) | x | x | x | x |  |
|  |  | Day 5 | before breakfast | x | x | x | x |  |
|  |  |  | after school (before eating) | x | x | x | x |  |
| 9 | House of Keto | Day 1 | before breakfast | 0.00 | 0.10 | 12.00 | x |  |
|  |  |  | after school (before eating) | 0.02 | 0.30 | 18.60 | x | No sensor - no lunch bolus |
|  |  | Day 2 | before breakfast | 0.00 | 0.10 | 15.60 | x | BGL 15.1 |
|  |  |  | after school (before eating) | x | x | x | x |  |
|  |  | Day 3 | before breakfast | 0.00 | 0.10 | 7.40 | x |  |
|  |  |  | after school (before eating) | 0.00 | 0.10 | 8.80 | x | BGL 9.5 - 8.6 |
|  |  | Day 4 | before breakfast | 0.00 | 0.10 | 9.30 | x | BGL 10.2 |
|  |  |  | after school (before eating) | x | x | x | x |  |
|  |  | Day 5 | before breakfast | 0.00 | 0.10 | 7.80 | x | BGL 7.5 |
|  |  |  | after school (before eating) | 0.00 | 0.00 | 4.80 | x |  |
|  |  | Day 6 | before breakfast | 0.00 | 0.10 | 5.80 | x | BGL 6.6 |
|  |  |  | after school (before eating) | 0.00 | 0.10 | 7.40 | x | BGL 7.7 |
|  |  | Day 7 | before breakfast | 0.00 | 0.00 | x | x |  |
|  |  |  | after school (before eating) | x | x | x | x |  |
|  |  | Day 8 | before breakfast | 0.00 | 0.00 | 8.10 | x | BGL 8.8 |
|  |  |  | after school (before eating) | x | x | x | x |  |
|  |  | Day 9 | before breakfast | 0.00 | 0.00 | 7.80 | x |  |
|  |  |  | after school (before eating) | 0.00 | 0.10 | 8.60 | x |  |
|  |  | Day 10 | before breakfast | 0.00 | 0.10 | 12.70 | x | BGL 14.4 |
|  |  |  | after school (before eating) | x | x | x | x |  |
| 10 | House of Keto | Day 1 | before breakfast | 0.00 | 0.00 | 7.60 | No |  |
|  |  |  | after school (before eating) | 0.00 | 0.00 | 5.70 | No |  |
|  |  | Day 2 | before breakfast | 0.00 | 0.00 | 6.10 | No |  |
|  |  |  | after school (before eating) | 0.00 | 0.00 | 4.20 | No |  |
|  |  | Day 3 | before breakfast | 0.00 | 0.00 | 7.80 | No |  |
|  |  |  | after school (before eating) | 0.00 | 0.00 | 9.50 | No |  |
|  |  | Day 4 | before breakfast | 0.00 | 0.00 | 8.20 | No |  |
|  |  |  | after school (before eating) | 0.00 | x | 7.80 | No |  |
|  |  | Day 5 | before breakfast | 0.00 | 0.10 | 12.00 | No |  |
|  |  |  | after school (before eating) | 0.00 | 0.10 | 10.50 | No |  |
|  |  | Day 6 | night | 0.00 | 0.20 | 17.00 | No | Just a random test because of high BGL |

*Note*. BGL= blood glucose level
